# Effects of the uremic toxin indoxyl sulphate on human microvascular endothelial cells

**DOI:** 10.1002/jat.4366

**Published:** 2022-07-25

**Authors:** Graziano Colombo, Emanuela Astori, Lucia Landoni, Maria L. Garavaglia, Alessandra Altomare, Maria C. Lionetti, Nicoletta Gagliano, Daniela Giustarini, Ranieri Rossi, Aldo Milzani, Isabella Dalle‐Donne

**Affiliations:** ^1^ Department of Biosciences (Department of Excellence 2018–2022) Università degli Studi di Milano Milan Italy; ^2^ Department of Pharmaceutical Sciences Università degli Studi di Milano Milan Italy; ^3^ Department of Biomedical Sciences for Health Università degli Studi di Milano Milan Italy; ^4^ Department of Life Sciences, Laboratory of Pharmacology and Toxicology University of Siena Siena Italy

**Keywords:** cardiovascular diseases, chronic kidney disease, gel‐free proteomic, HMEC‐1 cells, indoxyl sulphate

## Abstract

Indoxyl sulphate (IS) is a uremic toxin accumulating in the plasma of chronic kidney disease (CKD) patients. IS accumulation induces side effects in the kidneys, bones and cardiovascular system. Most studies assessed IS effects on cell lines by testing higher concentrations than those measured in CKD patients. Differently, we exposed a human microvascular endothelial cell line (HMEC‐1) to the IS concentrations measured in the plasma of healthy subjects (physiological) or CKD patients (pathological). Pathological concentrations reduced cell proliferation rate but did not increase long‐term oxidative stress level. Indeed, total protein thiols decreased only after 24 h of exposure in parallel with an increased Nrf‐2 protein expression. IS induced actin cytoskeleton rearrangement with formation of stress fibres. Proteomic analysis supported this hypothesis as many deregulated proteins are related to actin filaments organization or involved in the endothelial to mesenchymal transition. Interestingly, two proteins directly linked to cardiovascular diseases (CVD) in in vitro and in vivo studies underwent deregulation: COP9 signalosome complex subunit 9 and thrombomodulin. Future experiments will be needed to investigate the role of these proteins and the signalling pathways in which they are involved to clarify the possible link between CKD and CVD.

## Abstract 2

We exposed HMEC‐1 to the IS concentrations measured in the plasma of healthy subjects or CKD patients. Pathological concentrations reduced cell proliferation rate but did not increase long‐term oxidative stress level. Total protein thiols were decreased only after 24 h of exposure in parallel with an increase in Nrf‐2 protein expression. IS induced actin cytoskeleton rearrangement with formation of stress fibres. Proteomic analysis data suggest that IS could also act as an endothelial to mesenchymal transition stimulus.

## 1 INTRODUCTION

Chronic kidney disease (CKD) is a chronic and progressive disease with a world prevalence of 8% to 16%, and the WHO declared it as a public health problem, which is continually increasing (Jha et al., 2013). Patients are said to have CKD if they have abnormalities of kidney function or structure present for more than 3 months. CKD is diagnosed according two alternative or concomitant situations:

1. when a decreased kidney function is highlighted by a glomerular filtration rate (GFR) less than 60 ml/min (established for a reference man with 1.73 m^2^ body surface area) on at least 2 occasions 90 days apart (Webster et al., 2017);
2. when, independently on GFR, patients show markers of kidney damage (albumin/creatinine ratio [ACR] > 3 mg/mmol), urinary sediment abnormality, electrolyte abnormalities due to tubular disorders, renal histological abnormalities, structural abnormalities detected by imaging (e.g., polycystic kidneys and reflux nephropathy) or a history of kidney transplantation (Webster et al., 2017).

CKD is characterized by a reduced renal function, which worsens with the progression of the pathology, from Stage 1 to Stage 5 (Stenvinkel et al., 2008). As a result, patients with CKD show a pathological retention of various molecules, which normally are excreted by the kidneys (Vanholder et al., 2003). When these molecules induce side effects they are called uremic toxins.

The renal impairment predisposes to numerous complications, whose seriousness increases in parallel with the GFR decline. Among them, there are cardiovascular diseases (CVD), acute kidney injury, bone disorder, mineral balance disorder, hospitalization, anaemia, oxidative stress, chronic inflammation and dysbiosis (Stenvinkel et al., 2008). Dysbiosis is a feature of CKD patients, who show a quantitative and qualitative alteration of intestinal microflora. From the early stages of CKD, there is a change in composition and structure of the microbiota. Uremic patients show a higher number of *Enterobacteria* and *Enterococci* and a lower number of *Lactobacillaceae* and *Prevotellaceae* families (Vaziri et al., 2013). Haemodialysed (HD) patients present an overgrowth of aerobic bacteria, with the number of *Enterobacteria* and *Enterococci* species approximately 100 times higher than in healthy subjects. Regarding anaerobic bacteria, HD patients have a significantly lower number of *Bifidobacteria* and higher *Clostridium perfringens* (Hida et al., 1996).

Gut microbiota and CKD have a bidirectional relationship: kidney disease may disrupt microbiota balance and, at the same time, the unbalanced microbiota affects kidney disease progression and complications. About this last point, many recent studies suggest that toxic products generated by a dysbiotic gut microbiome may contribute to the progression of CKD and CKD‐related complications (Ramezani & Raj, 2014). In the colon, protein fermentation by intestinal bacteria generates several metabolites including ammonium, amines, thiols, phenols and indoles. Normally, they are in part eliminated through faeces and in part cleared by kidneys, but they accumulate in CKD and are called uremic toxins (Evenepoel et al., 2009). Because of the biological and clinical consequences of its accumulation, indoxyl sulphate (IS) is among the most studied metabolites in this context. It is associated with CKD progression, cardiovascular complications, alteration of bone‐mineral metabolism, insulin resistance and anaemia (Cigarran Guldris et al., 2017).

IS is a protein‐bound uremic toxin. It is a tryptophan metabolite produced by the gut microbiota: some microbes in the colon can convert dietary tryptophan in indole, which is absorbed into the blood circulation and reaches the liver, where it is oxidized and sulphated to form IS (Leong & Sirich, 2016). Normally, IS is cleared through renal tubular secretion, whereas in CKD patients it accumulates in blood and tissues, at least 90% bound to plasma proteins (Eloot et al., 2016).

IS has adverse effects mainly on kidneys, bones and cardiovascular system (Leong & Sirich, 2016). Regarding the nephrotoxicity, IS accelerates CKD progression inducing tubulointerstitial fibrosis and glomerular sclerosis (Niwa & Ise, 1994) as well as inflammation (Sun et al., 2013). About bone toxicity, IS appears to deteriorate bone mechanical proprieties (Iwasaki et al., 2013) and to induce skeletal resistance to parathyroid hormone (Nii‐Kono et al., 2007). Overall, most studies are about the correlation between IS and CVD, because IS seems to play an important role in the progression of CVD observed in CKD patients (Gao & Liu, 2017). Indeed, patients with CKD are at higher risk of CVD than the normal population. For example, patients with Stage 5 CKD, or end‐stage renal disease, have a cardiovascular mortality 10‐ to 30‐fold higher than healthy subjects (Jha et al., 2013). In this context, also uremic toxins play a role: in particular, IS seems to contribute to higher risk of chronic heart failure, arrhythmia, coronary calcification and atherosclerotic vascular diseases (Gao & Liu, 2017). These correlations may depend mainly on the enhanced oxidative stress induced by IS in the myocardium and vasculature (Ito & Yoshida, 2014; Lekawanvijit et al., 2012). Impairment of endothelial function has many consequences, which can lead to vascular diseases, such as atherosclerosis (Endemann & Schiffrin, 2004). IS causes endothelial dysfunction through several mechanisms, mostly involving an imbalance between pro‐oxidant and antioxidant mechanisms (Dou et al., 2007; Yu et al., 2011), inflammation pathways (Adelibieke et al., 2014; Pletinck et al., 2013; Watanabe et al., 2013), dysfunctions in cell proliferation and wound repair capabilities (Dou et al., 2004; Yu et al., 2011), thus compromising endothelial barrier functions (Peng et al., 2012).

**FIGURE 1:**
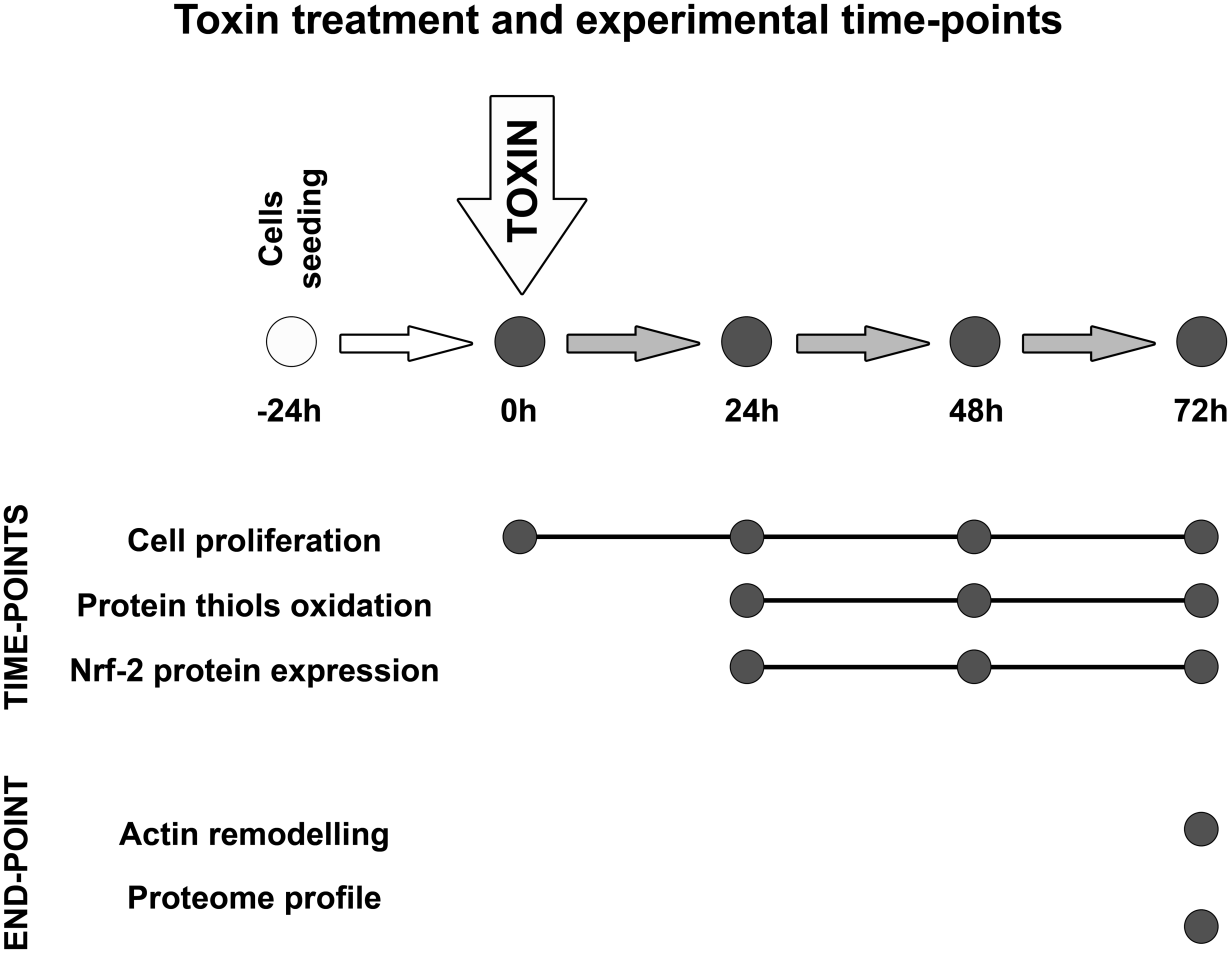
Study design: The study shown in this paper can be divided into two experimental approaches aimed to evaluate the acute effects of IS (cell proliferation, protein thiols oxidation and Nrf‐2 protein expression) and the 3‐day treatment effects (cytoskeleton organization and proteome profile).

Most studies to assess IS effects on endothelium have been performed on human umbilical vein endothelial cells (HUVECs), testing concentration much higher than those measured in vivo (Vanholder et al., 2014). In this study, we assessed some effects of pathophysiological concentrations of IS on human microvascular endothelial cells because the microcirculation is the principal seat of exchanges between the circulation and body tissues. Briefly, the design of the study can be divided into two experimental approaches (Figure 1):

1. time‐point analysis: we followed over time the evolution of the treatment to explore the acute effects of IS (cell proliferation, protein thiols oxidation and Nrf‐2 protein expression);
2. end‐point analysis: we examined the effects of a three‐day treatment to investigate the possible ‘chronic’ effects on cytoskeleton organization and proteome profile.

## 2 MATERIALS AND METHODS

### 2.1 Cell line and solutions for cell maintenance

Human dermal microvascular endothelial cells‐1 (HMEC‐1) line was obtained from the American Type Culture Collection (Manassas, VA, USA) and grown in plates with MCDB 131 Medium (Sigma‐Aldrich, Milan, Italy), supplemented with 10% foetal bovine serum (Euroclone, Milan, Italy), 2 mM l‐glutamine, 100 U/ml penicillin, 100 μg/ml streptomycin, 10 ng/ml epidermal growth factor and 0.1 μg/ml hydrocortisone (Sigma‐Aldrich, Milan, Italy). Cell cultures were maintained at 37°C with 5% CO_2_ and passaged every 3–4 days. For experiments, HMEC‐1 cells were cultured in the presence or absence of different concentrations of IS potassium salt (Sigma‐Aldrich, Milan, Italy) for 24, 48 or 72 h.

### 2.2 Treatment of HMEC‐1 with IS

HMEC‐1 cells were seeded at a concentration of 15,000 cells/cm^2^ and let grown for 24 h, at 37°C, with 5% CO_2_. Then, a half of the medium was removed and replaced with an equal volume of solution with or without IS. This expedient is necessary because HMEC‐1 release growth factors in the medium and a complete replacement of the culture medium would slow down cell growth. IS solutions were prepared dissolving IS in phosphate‐buffered saline (PBS), obtaining a mother solution with a concentration of 10 g/L, which was diluted in complete medium (prepared as described above) at the following concentrations: 1, 50 and 100 mg/L. These concentrations are double to the desired ones, because only a half of the medium was changed. IS solutions were filtered through a syringe with a 0.22‐μm pore‐sized filter to remove bacteria and particulate and added to the cell culture at the final concentrations of 0.5, 25 and 50 mg/L IS. Control cells were treated in a similar way, without the toxin. Therefore, all the treatment solutions contained the same volume of PBS and they differed only for the presence or absence of IS. The treatment lasted 24, 48 or 72 h, without changing the medium.

### 2.3 Proliferation assay

Sulforhodamine B (SRB) assay is a colorimetric test that allows quantifying cellular protein content and it is largely used to indirectly evaluate cell proliferation (Orellana & Kasinski, 2016; Skehan et al., 1990; Vichai & Kirtikara, 2006; Voigt, 2005). Briefly, cells were seeded and treated as described above in 24‐multiwell plates. At each time point, cells were fixed with 50% trichloroacetic acid (Sigma‐Aldrich, Milan, Italy; T6399) for 2 h at 4°C and washed five times with Milli‐Q water; 0.04% (w/v) SRB dye (Sigma‐Aldrich, Milan, Italy; S1402), dissolved in 1% acetic acid, was added to each well and incubated at room temperature for 30 min; then, each well was washed four times with 1% (v/v) acetic acid and left to air‐dry at room temperature. Finally, 1.2 ml of 10 mM Tris base solution (pH 10.5) was added to each well, and the plate was shaken on an orbital shaker for 10 min to solubilize the protein‐bound dye. The absorbance at 490 nm was detected using a multimode microplate reader (EnSight Plate Reader, PerkinElmer).

### 2.4 Quantification of proteins thiols

Cell protein extracts were obtained by lysing cells with ice‐cold lysis buffer (50 mM Tris–HCl, pH 7.4, 150 mM NaCl, 1% Triton X‐100, 0.1% SDS and 0.5% sodium deoxycholate supplemented with protease inhibitors [Sigma‐Aldrich, Milan, Italy, P8340]), followed by incubation on ice for 30 min and centrifugation at 10,000 *g* for 10 min at 4°C to remove cell debris. Protein concentration in supernatants was determined by bicinchoninic acid (BCA) protein assay. Protein thiol groups were detected by a biotin‐maleimide assay. Briefly, 40 mM biotin‐maleimide stock solution was prepared in dimethyl sulphoxide and stored at −20°C; 1 mg/ml of protein was incubated with 75 μM biotin‐maleimide solution for 1 h at room temperature and then mixed with Laemmli sample buffer (2% SDS, 20% glycerol, 125 mM Tris–HCl and pH 6.8), heated for 5 min at 90°C and separated on 12% SDS‐PAGE Stain‐free gel (Bio‐Rad) (Hill et al., 2009). Separated proteins were electroblotted onto a low‐fluorescence polyvinylidene difluoride (LF‐PVDF) membrane. LF‐PVDF membrane was washed with PBST (10 mM Na‐phosphate, pH 7.2, 0.9% (w/v) NaCl and 0.1% (v/v) Tween‐20 [Sigma‐Aldrich, P9416]) (Hill et al., 2009) and blocked for 1 h in 5% (w/v) non‐fat dry milk in PBST. After washing three times with PBST for 5 min, the biotin tag was probed by a 2‐h incubation with 5% non‐fat dry milk/PBST containing streptavidin‐HRP (1:5000 dilution, Cytiva). Biotinylated proteins were visualized by ECL detection (Bio‐Rad, 1705061) using the ChemiDoc Touch Imaging System (Bio‐Rad). ECL signals were normalized on LF‐PVDF stain‐free signals (Rivero‐Gutiérrez et al., 2014).

### 2.5 Western blot

Proteins from cell extracts were separated and transferred to LF‐PVDF membrane as described above. After washing three times with TBST for 5 min, the membrane was incubated for 2 h with 5% non‐fat dry milk/TBST containing the following primary antibodies: anti‐Nrf‐2 (1:1000, Enzo Life Sciences), anti‐actin (1:2000, Abcam), anti‐tubulin (1:40,000, Abcam) and anti‐VE‐cadherin (1:1000, Cell Signaling Technology). The membrane was washed three times with TBST for 5 min and then incubated with the following secondary antibodies, respectively: anti‐rabbit (1:10,000), anti‐mouse (1:10,000), anti‐rabbit (1:20,000) and anti‐rabbit (1:20,000). Proteins of interest were visualized by ECL detection (Bio‐Rad, 1705061) using the ChemiDoc Touch Imaging System (Bio‐Rad). ECL signals were normalized on LF‐PVDF stain‐free signals (Rivero‐Gutiérrez et al., 2014) and using tubulin as housekeeping protein.

### 2.6 Immunofluorescence

HMEC‐1 cells were cultured on 12‐mm‐diameter round coverslips, grown on 24‐well culture plates and treated with IS as described above. At each time point, cells were washed in PBS, fixed in 4% paraformaldehyde in PBS containing 2% sucrose for 10 min at room temperature, post‐fixed in 70% ethanol and stored at −20°C until use. For microtubules analysis, cells were washed in PBS three times, incubated 5 min at room temperature with 0.1% Triton X‐100/PBS and blocked with 1% bovine serum albumin (BSA) in PBS for 1 h. Cells were then incubated with the primary monoclonal anti‐tubulin antibody (1:300 [Abcam], diluted in 0.5% BSA/PBS) at 4°C overnight. The next day cells were washed four times with PBS, incubated for 1 h in the dark with the secondary antibody, an anti‐rabbit TRITC‐conjugated (Abcam) diluted 1:200 in 0.5% BSA/PBS, and washed extensively in PBS. For microfilament detection, cells were then incubated for 1 h in the dark with fluorescein (FITC)‐phalloidin 1:1000 (Abcam) in 1% BSA/PBS. After the labelling procedure was completed, the coverslips were incubated for 10 min with 4′6‐diamidino‐2‐phenylindole (DAPI) and mounted onto glass slides using Mowiol mounting medium. Fixed cells were imaged with a ViCo confocal microscope (Nikon) and TCS NT confocal laser scanning microscope (Leica).

### 2.7 Quantitative proteomic analysis of HMEC‐1 cells after a 72‐h treatment with IS

HMEC‐1 cells were seeded and treated with IS for 72 h as described above, without changing the medium. After removal of medium and three washes with PBS, cell protein extracts were obtained by lysing cells with the following lysis buffer: 8 M urea, 100 mM Tris–HCl, pH 8.5, protease inhibitors (Sigma‐Aldrich, P8340). Each lysate was incubated for 30 min at room temperature and centrifuged at 14,000 *g* for 30 min at 4°C to remove cell debris. Protein concentration in supernatants was determined by Bradford protein assay. To check the integrity of extracted proteins, part of the lysate was mixed with Laemmli sample buffer, heated for 5 min at 90°C and separated on 12% SDS‐PAGE Stain‐free gel (Bio‐Rad) (Hill et al., 2009). Protein gel was acquired using the ChemiDoc Touch Imaging System (Bio‐Rad). The rest of each lysate was used to perform tryptic digestion of proteins. Ten micrograms of proteins was mixed in 36 μl of 50 mM ammonium bicarbonate (AMBIC) dissolved in MS‐grade water (Sigma‐Aldrich). pH was checked to ensure that it was around 8–8.5. Then, 5 mM dithiothreitol (DTT, diluted in AMBIC) was added, and samples were incubated in a Thermomixer at 600 rpm, 52°C for 30 min. At this point, 15 mM iodoacetamide (IAM, diluted in AMBIC) was added, and the samples were incubated in a Thermomixer at 600 rpm, at room temperature for 20 min, in the dark; 0.5 μg trypsin in 50 mM acetic acid was added (after activation for 15 min at 30°C) respecting a ratio 1:20 trypsin:protein. Samples were incubated in a Thermomixer at 600 rpm, 37°C overnight. The day after, 2 μl of 50% trifluoroacetic acid (diluted in MS‐grade water) was added and the pH was checked to ensure that it was lower than 2.

### 2.8 High‐resolution mass spectrometry analysis (nLC‐MSMS)

Tryptic peptides were analysed at UNITECH OMICs (University of Milano, Italy) using a Dionex Ultimate 3000 nano‐LC system (Sunnyvale CA, USA) connected to an Orbitrap Fusion™ Tribrid™ Mass Spectrometer (Thermo Scientific, Bremen, Germany) equipped with a nanoelectrospray ion source. Peptide mixtures were preconcentrated onto an Acclaim PepMap 100—100 mm, 2 cm C18 and separated on EASY‐Spray column, 15 cm, 75 mm ID packed with Thermo Scientific Acclaim PepMap RSLC C18, 3 mm, 100 Å. The temperature was set to 35°C, and the flow rate was 300 nl/min. Mobile phases were the following: 0.1% formic acid (FA) in water (solvent A) and 0.1% FA in water/acetonitrile (solvent B) with 2/8 ratio. Peptides were eluted from the column with the following gradient: 4% to 28% of B for 90 min and then 28% to 40% of B in 10 min and to 95% within the following 6 min to rinse the column. Column was re‐equilibrated for 20 min. Total run time was 130 min. One blank was run between triplicates to prevent sample carryover. MS spectra were collected over an *m/z* range of 375–1500 Da at 120,000 resolutions, operating in the data‐dependent mode, cycle time 3 s between master scans. HCD was performed with collision energy set at 35 eV. Each sample was analysed in three technical triplicates. LTQ raw data were searched against a protein database using SEQUEST algorithm in Proteome Discoverer software version 2.2 (Thermo Scientific) for peptide/protein identification. The searches were performed against Uniprot KnowledgeBase (KB) (taxonomy *Homo sapiens*). The minimum peptide length was set to six amino acids, and enzymatic digestion with trypsin was selected, with maximum two missed cleavages. A precursor mass tolerance of 8 ppm and fragment mass tolerance of 0.02 Da were used; acetylation (N‐term) and oxidation (M) were used as dynamic modifications and carbamidomethylation (C) as static modification. The false discovery rates (FDRs) at the protein and peptide level were set to 0.01 for highly confident peptide‐spectrum matches and 0.05 for peptide‐spectrum matches with moderate confidence. We considered only proteins with a score of coverage >2% with at least two identified peptides. Differences in abundance ratio (AR) of proteins between control and treated samples were considered only with at least a twofold change and with a standard deviation between replicates less than 20%.

## 3 RESULTS

### 3.1 IS alters growth rate of HMEC‐1 cells

**FIGURE 2:**
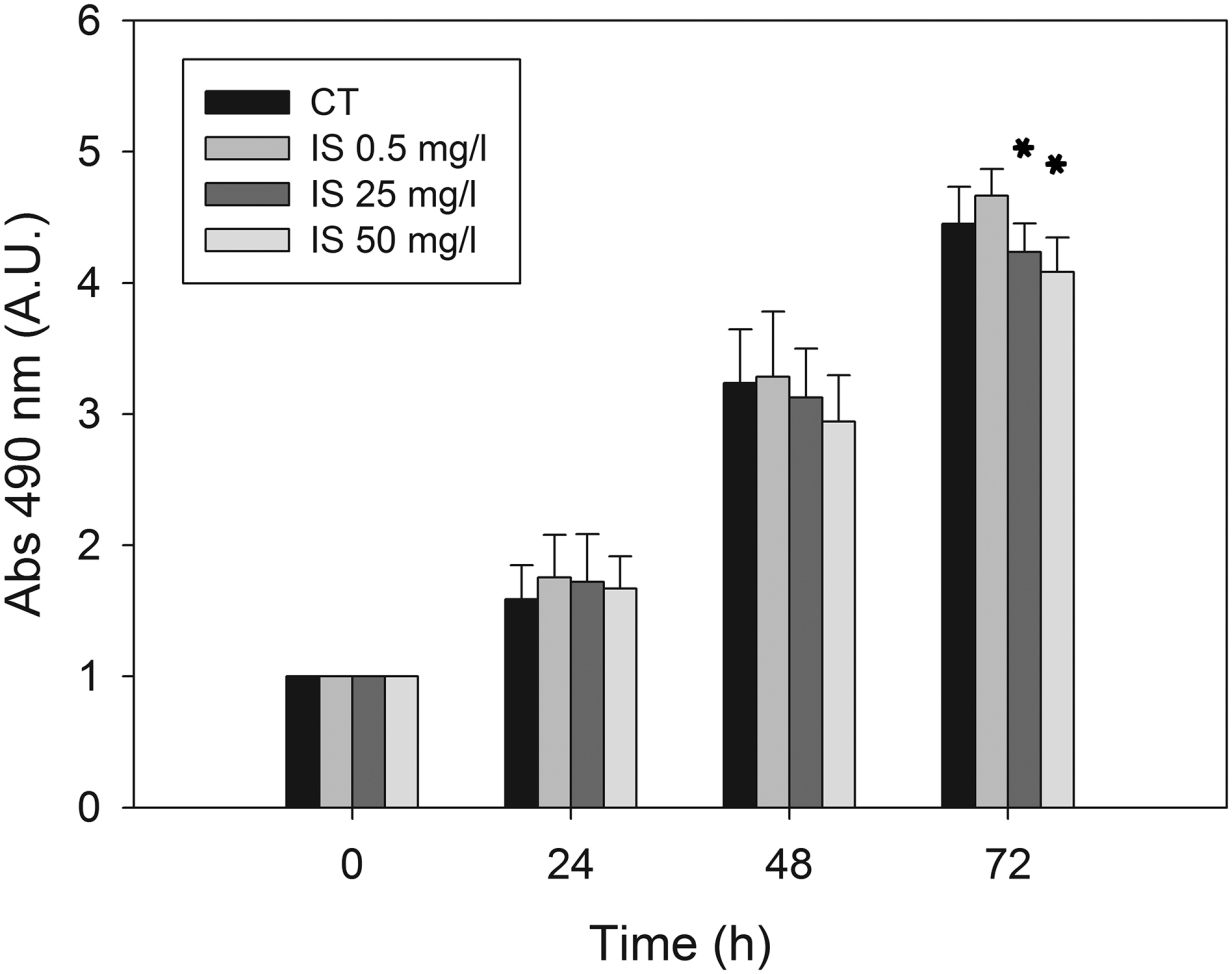
Effect of IS on HMEC‐1 cell proliferation measured by SRB assay: Histograms showing the mean absorbance measured at 490 nm in control cells and cells treated with 0.5, 25 or 50 mg/L of IS for 0, 24, 48 or 72 h. Data are expressed as the mean ± SD of four independent experiments. *p < 0.05.

The growth of cultured HMEC‐1 cells was followed up to 72 h. By using SRB assay (Figure 2), we observed a statistically significant reduction in the cell proliferation rate (*p* < 0.05) only after 72‐h exposure to pathological IS concentrations (25 and 50 mg/L). Control HMEC‐1 cells and HMEC‐1 cells treated with the physiological concentration (0.5 mg/L) grew exponentially over 3 days, whereas cells treated with IS 25 and 50 mg/L IS showed a progressive growth reduction over time.

### 3.2 IS induces oxidative stress and oxidative stress‐responses in HMEC‐1 cells

**FIGURE 3:**
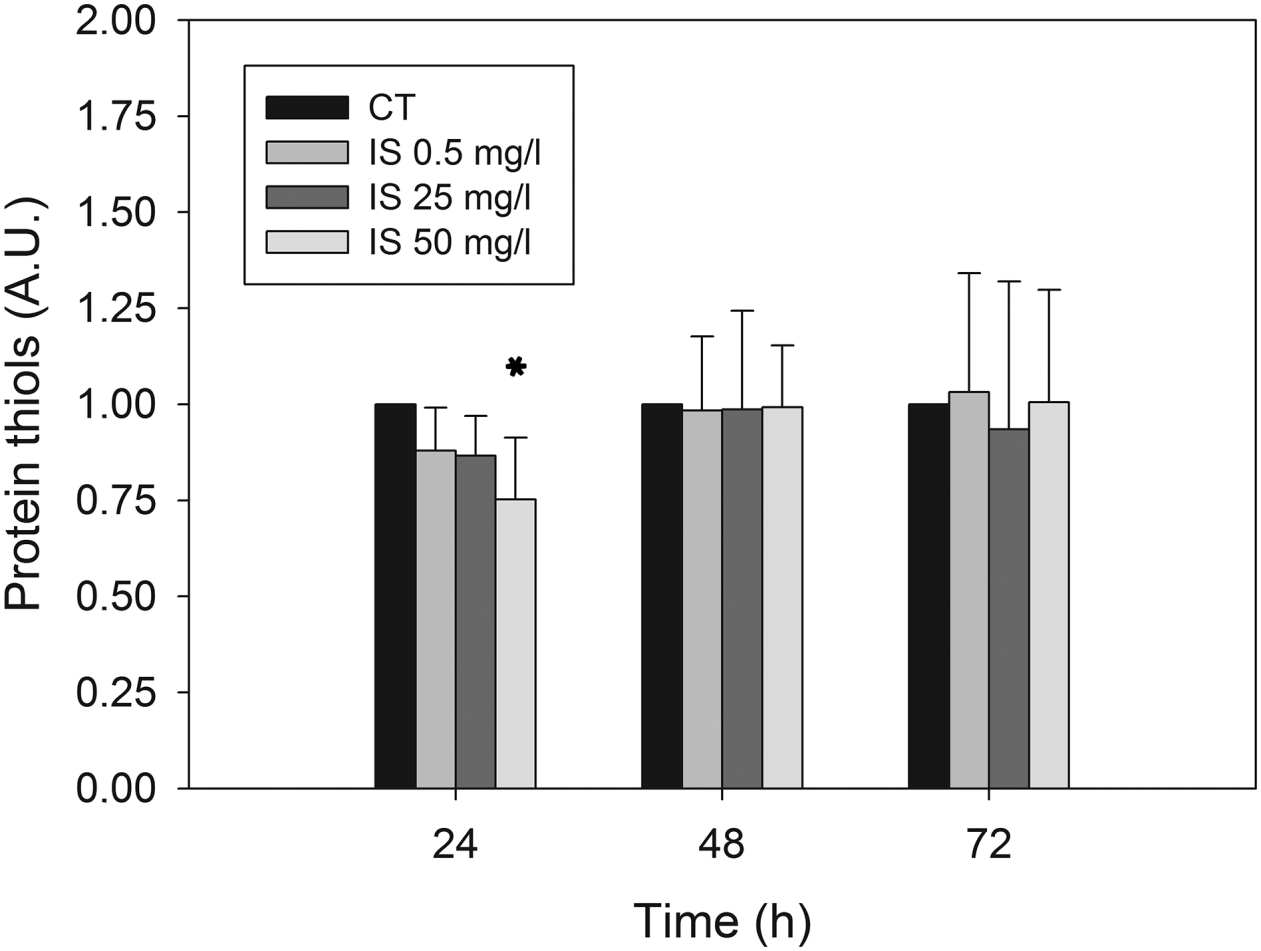
Effect of IS on the total amount of protein thiols in HMEC‐1 cells: Histograms showing protein thiol level in control cells and cells treated with 0.5, 25 or 50 mg/L IS for 0, 24, 48 or 72 h. Data are expressed as the mean ± SD of three independent experiments. *p < 0.05.

**FIGURE 4:**
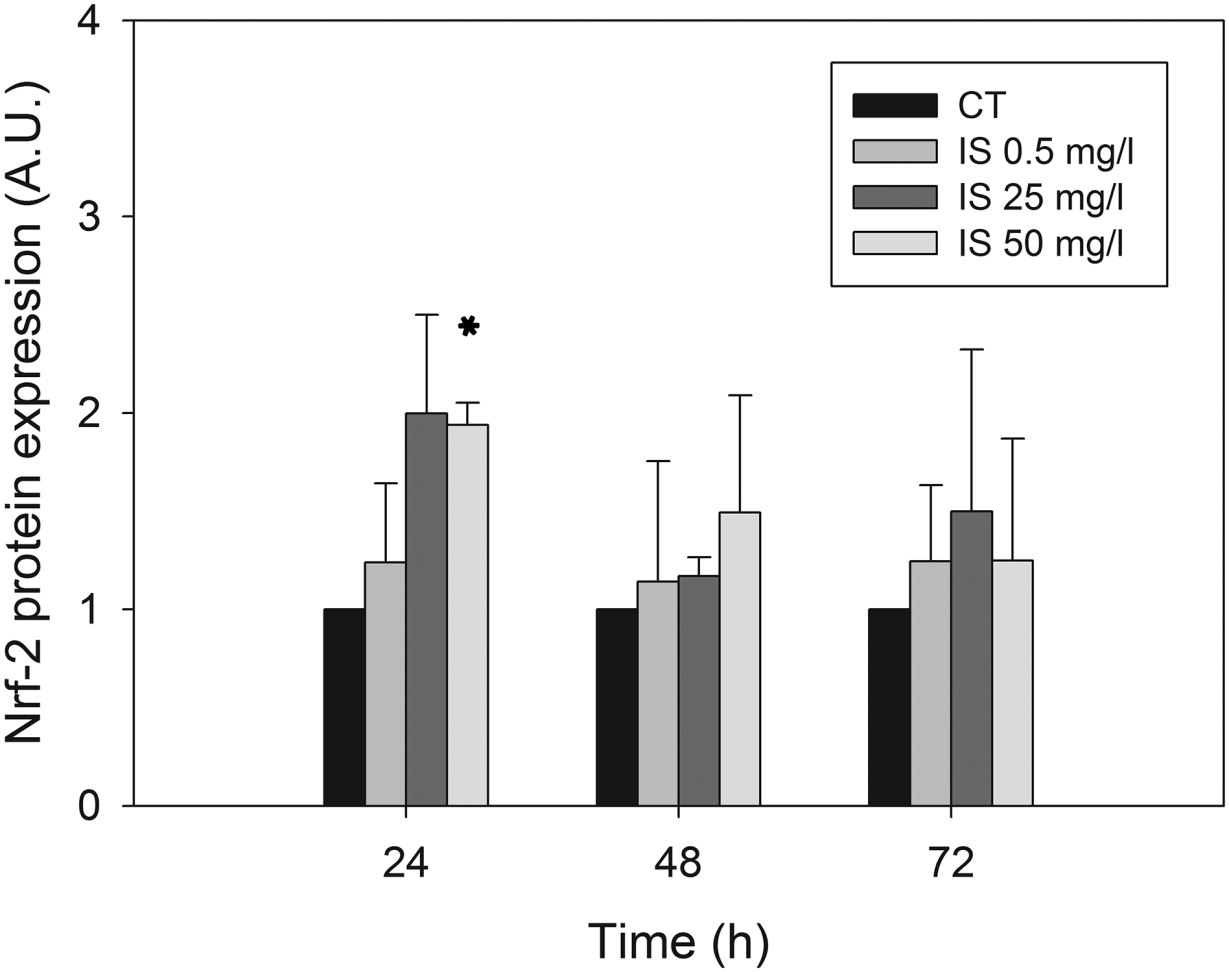
Effects of IS on Nrf‐2 expression in HMEC‐1 cells: Histograms showing the Nrf‐2 level measured in control cells and cells treated with 0.5, 25 or 50 mg/L IS for 0, 24, 48 or 72 h. Data are expressed as the mean ± SD of three independent experiments. *p < 0.05.

Emerging evidence from cellular and animal model studies (Edamatsu et al., 2018; Ji et al., 2018; Yang et al., 2015) as well as clinical studies in CKD patients (Fujii et al., 2011; Gao & Liu, 2017) reveal that IS induces oxidative stress. We evaluated pro‐oxidant effects of IS in HMEC‐1 cells assessing, by Western immunoblotting, protein carbonylation and oxidation of protein thiols as oxidative stress biomarkers in whole‐cell lysates. We did not find any difference in protein carbonylation throughout treatment at all concentrations tested (data not shown). Differently, we found a moderate, statistically significant and concentration‐dependent decrease in the total amount of protein thiols after 24 h of treatment (Figure 3). It is known that oxidative stress can lead to the formation of unwanted disulphide bonds in the cytoplasm, thus reducing the total amount of thiols. This event can lead to impaired protein function. To face this, cells have several mechanisms to increase the intracellular levels of thiols (Deneke, 2000). Notably, intracellular increase in thiol levels is strongly associated with increased tolerance to an oxidant stress (Deneke, 2000) because they act as extraordinarily efficient antioxidants protecting cells against consequences of damage induced by reactive oxygen species (ROS) (Włodek, 2002). As we detected significant decrease in the intracellular thiol concentration only after 24 h of cell treatment with IS, we evaluated Nrf‐2 expression by Western blot to assess eventual recovery mechanisms. Indeed, Nrf‐2 is a transcription factor whose activation is induced by oxidative stress (Ma, 2013). As shown in Figure 4, we found a significant increase in the level of Nrf‐2 in HMEC‐1 cells treated with IS 50 g/L for 24 h (*p* < 0.01).

### 3.3 IS induces stress fibre formation

**FIGURE 5:**
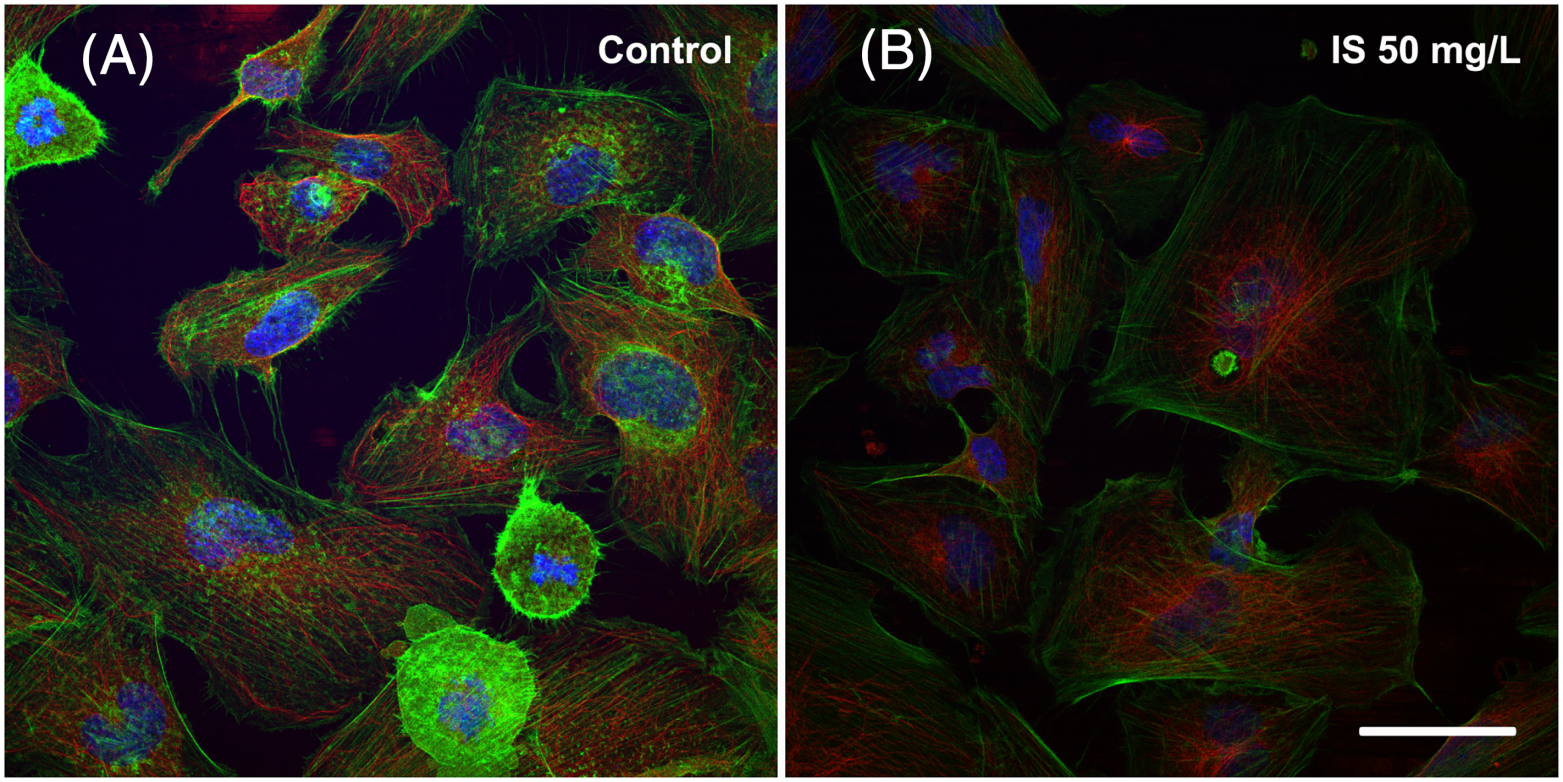
Effects of IS on the cytoskeleton organization of HMEC‐1 cells: (A) On the left, control cells observed at 72 h after the start of the treatment. (B) On the right, cells treated with 50 mg/L IS for 72 h. Images were acquired with TCS NT confocal laser scanning microscope (Leica). Green: microfilaments; red: microtubules; blue: DAPI used for nuclear staining. Bar 25 μm

**FIGURE 6:**
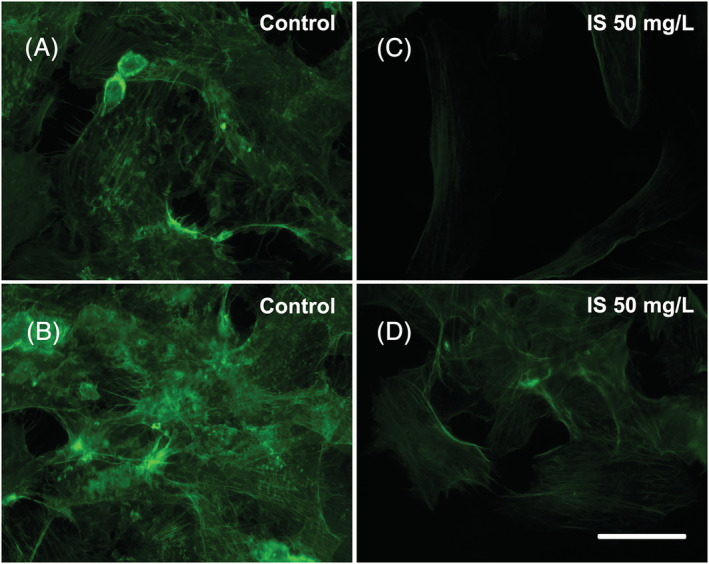
Effects of IS on the actin filament organization: (A,B) On the left, control HMEC‐1 cells observed at 72 h after the start of the treatment. (C,D) On the right, HMEC‐1 cells treated with 50 mg/L IS for 72 h. Images were acquired with ViCo confocal microscope (Nikon). Bar 25 μm

IS is reported to modify the cytoskeleton organization and to disrupt cell junctions, contributing to compromise endothelial barrier function (Peng et al., 2012). We evaluated the expression of cytoskeletal and tight junction proteins in whole‐cell lysates by Western immunoblotting. We found no significant differences in either VE‐cadherin or beta‐catenin expression (data not shown) as well as in actin or tubulin expression (data not shown). However, we found some differences looking at the cytoskeleton organization by immunofluorescence (Figure 5). Control cells showed randomized organization of actin filaments, while, after treatment with 50 mg/L IS for 72 h, actin filaments appeared organized in prominent parallel‐oriented stress fibres (Figure 6), as observed previously by others (Peng et al., 2012). Stress fibres were not observed in other time points or HMEC‐1 cells treated with lower concentrations of IS (data not shown).

### 3.4 IS weakly affects intracellular protein expression and medium‐released protein abundance

**FIGURE 7:**
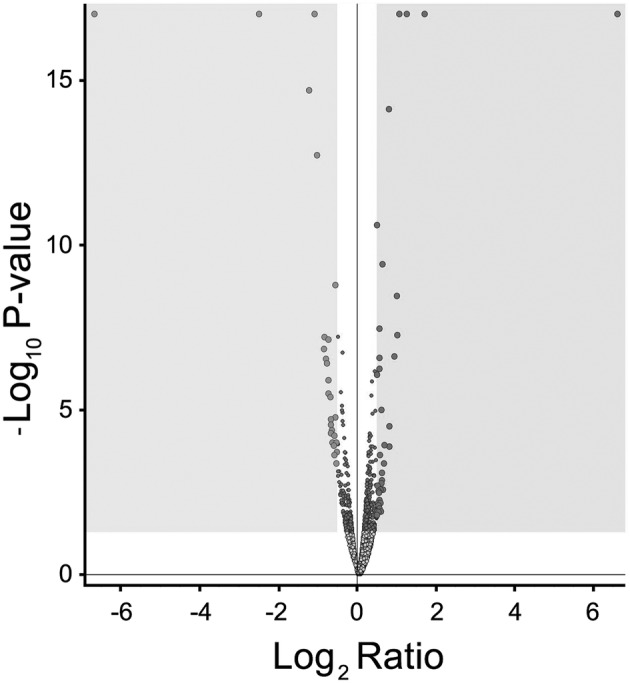
Volcano plot: Volcano plot of differentially expressed proteins between HMEC‐1 cells treated with 0.5 mg/L IS and HMEC‐1 cells treated with 50 mg/L of IS, for 72 h. Down‐regulated proteins are in light grey in the left grey box. Up‐regulated proteins are in dark grey in the right grey box.

**FIGURE 8:**
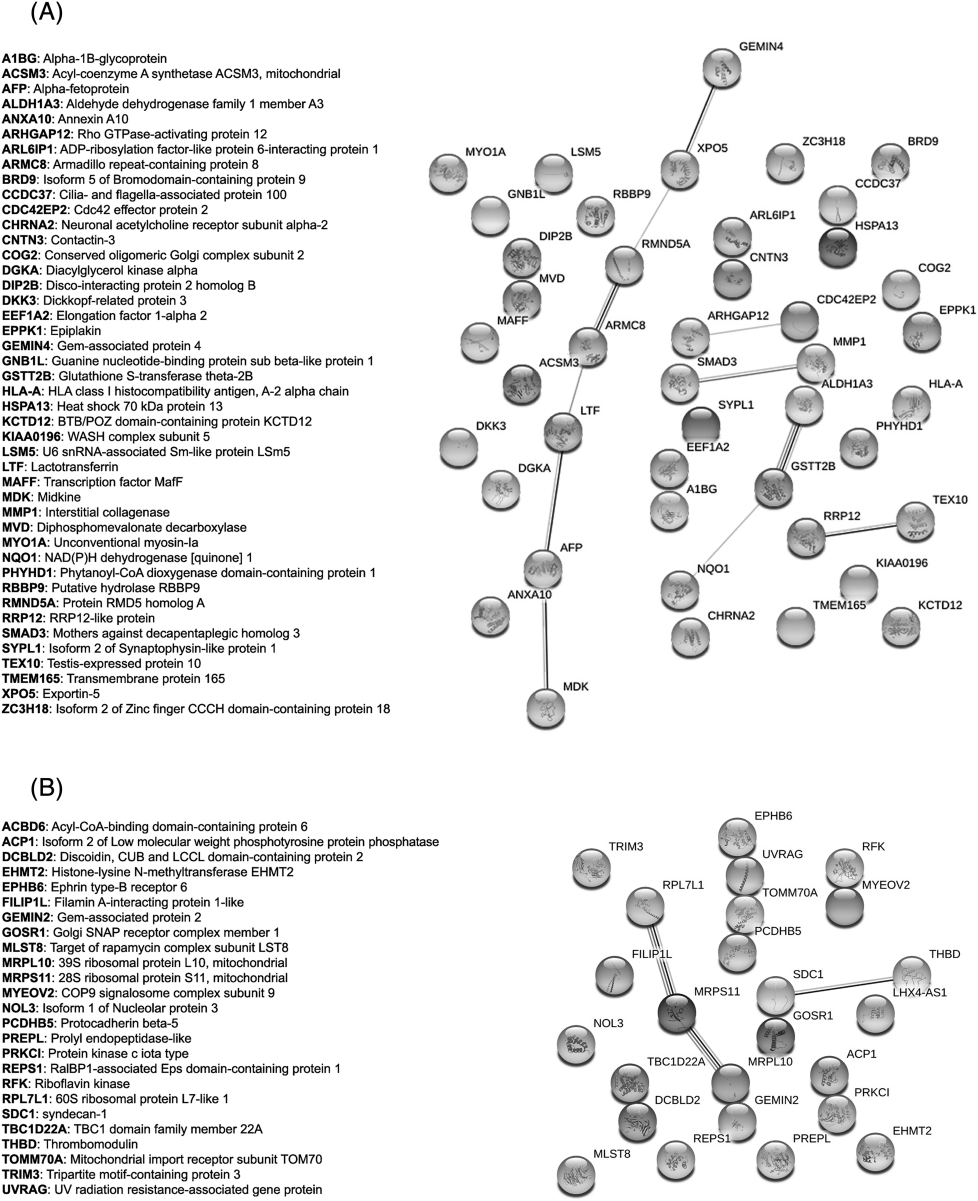
Network of the regulated proteins obtained with the software String: (A) The network includes the proteins that emerged as up‐regulated in HMEC‐1 cells treated with 50 mg/L IS compared with those treated with 0.5 mg/L IS, at 72 h after the start of the treatment. (B) The network includes the proteins that emerged as down‐regulated in HMEC‐1 cells treated with 50 mg/L IS compared with those treated with 0.5 mg/L of IS, at 72 h after the start of the treatment.

**FIGURE 9:**
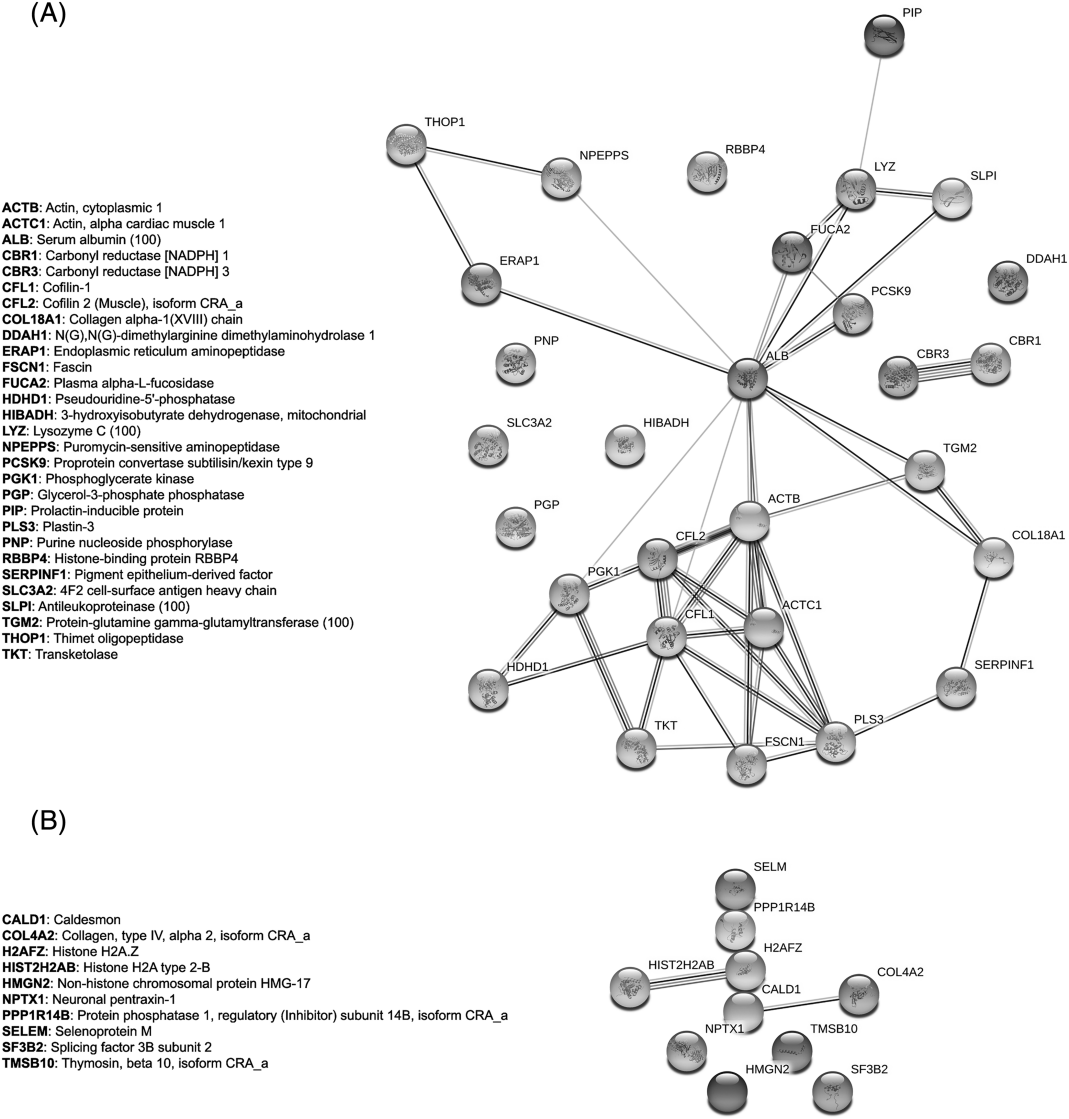
Network of the regulated proteins obtained with the software String: (A) The network includes the proteins that emerged as up‐regulated in the medium of HMEC‐1 cells treated with 50 mg/L IS compared with those treated with 0.5 mg/L IS, at 72 h after the start of the treatment. (B) The network includes the proteins that emerged as down‐regulated in the medium of HMEC‐1 cells treated with 50 mg/L IS compared with those treated with 0.5 mg/L of IS, at 72 h after the start of the treatment.

For the proteomic analysis, we compared intracellular protein expression (Figure 8A,B) and medium‐released protein (Figure 9A,B) abundance in HMEC‐1 cells treated with 0.5 mg/L IS (mean physiological concentration measured in healthy subjects) and HMEC‐1 cells treated with 50 mg/L IS (higher concentration measurable in CKD patients). We did not compare control and treated HMEC‐1 cells because the control condition, that is, endothelial cells exposed to no concentration of IS, does not occur physiologically. The volcano plot (Figure 7) from intracellular proteome comparison shows that only a few proteins resulted to be up‐regulated or down‐regulated when comparing cells treated for 72 h with 0.5 versus 50 mg/L.

In detail, considering intracellular proteome analysis, only 44 and 25 proteins underwent up‐regulation and down‐regulation, respectively. Protein modulation after a 3‐day treatment with pathological IS concentration was higher than 1.5‐fold increase/decrease. Up‐regulated proteins ranged between 1.5‐ and 3.3‐fold change (elongation factor 1‐alpha 2), whereas down‐regulated proteins ranged between 1.5‐ and 5.6‐fold change (thrombomodulin). Similarly, considering medium‐released proteome analysis, only 29 and 10 proteins underwent up‐regulation and down‐regulation, respectively. Protein release in the medium after a 3‐day treatment with pathological IS concentration was modulated with a fold increase/decrease change higher than 1.5.

In order to see whether these proteins were linked together according to their functions, we performed a STRING analysis obtaining the functional network of up‐regulated (Figures 8A and 9A) and down‐regulated proteins (Figures 8B and 9B). The four networks do not have significantly more interactions than expected, according to STRING lambda calculation. This means that our sets of proteins are composed by an apparently random collection of proteins that are not strictly connected or whose interactions are not yet known by STRING database according to available data.

The functional investigation (protein–protein interaction networks' analysis) (Figures 8A,B and 9A,B) did not reveal any significantly activated or inactivated pathways; therefore, we proceeded with the nonautomated interpretation by gathering information from the most recent literature.

## 4 DISCUSSION

In this study, we have tried to better clarify some of the side effects of IS when cells are exposed to pathophysiological concentrations of this toxin. We used an endothelial cell line from the microvasculature, because IS is strongly correlated with CVD (Gao & Liu, 2017) and the literature lacks works evaluating IS effects in the microcirculation, even though it is the principal seat of exchanges between circulation and tissues.

IS caused a significant reduction in the proliferation rate (*p* < 0.05) of HMEC‐1 cells exposed for 72 h to pathological IS concentrations (25 and 50 mg/L), in agreement with other studies in which comparable IS concentrations were tested on HUVEC (Dou et al., 2004; Yu et al., 2011). Some authors also observed cell senescence, decreased nitric oxide production and oxidative stress, suggesting that IS‐induced oxidative stress could be, therefore, responsible for the inhibition of cell proliferation (Yu et al., 2011). Considering that the ability of cells to proliferate, as well as their ability to migrate, play an important role in the healing of endothelial injury, Dou et al. (2004) evaluated the wound repair ability of HUVEC treated with IS and found it inhibited. We tried to test whether HMEC‐1 cells exposed to IS also showed inhibition of wound repair, but we were not able to obtain optimal cells adhesion on plastic support to perform the scratch assay.

Patients with CKD show high oxidative stress level, which is strongly correlated with two important risk factors for mortality: chronic inflammation and CVD (Colombo et al., 2015). Uremic toxins contribute to oxidative stress and IS has been extensively correlated with this problem. IS‐induced oxidative stress exerts side effects on the skeletal system (Liu et al., 2018), the kidneys (Chao & Chiang, 2015) and the cardiovascular system (Gao & Liu, 2017). In the cardiovascular system, IS seems to enhance oxidative stress both in the myocardium and the blood vessels (Ito & Yoshida, 2014; Lekawanvijit et al., 2012). An in vitro study on HUVEC reports that a 5‐h IS incubation stimulates ROS production, increases NAD(P)H oxidase activity and decreases glutathione levels, thus increasing oxidative stress by both promoting pro‐oxidant and inhibiting antioxidant activities (Dou et al., 2007). Another study on HUVEC hypothesizes that IS‐induced oxidative stress could arise from NADPH oxidase and mitochondrial respiratory chain complex activities and that it could be responsible for endothelial dysfunctions seen after 24 or 48 h of IS exposure (Yu et al., 2011). It is interesting to note that in the latter study ROS production was only measured up to 12 h of exposure to IS, during which even the highest concentration of IS tested (125 mg/L, more than twofold higher than the highest concentration we used in the present study, i.e., 50 mg/L) caused an increase in ROS that reached a plateau in the first few hours. Another study reported an increase in ROS production in HUVEC after exposure to 125 mg/L of IS for 2 min, mainly attributable to the activity of NADPH oxidase isoform 4 (Nox4), the most dominant isoform in the kidney (Masai et al., 2010).

In our study, we evaluated two well‐recognized oxidative stress biomarkers after 24, 48 and 72 h of HMEC‐1 cell exposure to IS: protein carbonylation and the total amount of protein thiols (Dalle‐Donne et al., 2006). For both biomarkers, we found no significant differences between control and treated cells except in one case. These results appear to be in contrast with the other studies mentioned above, but we can speculate that these differences could depend on different time points. In most of the studies in the literature, IS effects are assessed in the first hours of exposure, whereas we tried to mimic chronic exposure by assessing IS effects over 3 days. During this period of time, cells may activate some recovery system, and this could explain the low impact on oxidative stress biomarkers observed. In this regard, 24 h after exposure of HMEC‐1 cells to IS, we found a decrease in the total amount of protein thiols that was significant only at 50 mg/L IS; after 48 and 72 h, the amount of protein thiols in control cells and in cells treated with the three IS concentrations was similar. We also found a significant increase in the level of Nrf‐2 in HMEC‐1 cells treated with IS 50 mg/L for 24 h (*p* < 0.01). Nrf‐2 activates genes that encode for phase II detoxifying enzymes and antioxidant enzymes, which counteract oxidative stress (Stockler‐Pinto et al., 2014). Therefore, we can assume that Nrf‐2 activation in HMEC‐1 treated with IS could be one of the recovery mechanisms that can reduce oxidative stress, thus justifying the homogeneity in the levels of oxidative stress biomarkers at 48 and 72 h. Reduced expression of Nrf‐2 in peripheral blood mononuclear cells from 20 haemodialysed patients (Stockler‐Pinto et al., 2014) and down‐regulation of Nrf‐2 expression in HK‐2 cells treated with IS (Bolati et al., 2013) are reported in the literature. However, these studies are not comparable with ours, as the former was conducted in vivo, while the latter was performed on a kidney cell line. Therefore, more work is needed to better elucidate the relationship between Nrf‐2 and IS in endothelial cells.

Other events that may compromise endothelial barrier function are cytoskeleton remodelling and disruption of intercellular junctions. A treatment lasted 24 h with 50–250 mg/L IS modified the shape of bovine pulmonary artery endothelial cells (BPAECs): while control cells had cobblestone‐like shape, with actin organized in randomized arrays, cells treated with IS were elongated, like fibroblasts, with actin organized in parallel‐oriented stress fibres (Peng et al., 2012). We observed a similar behaviour in HMEC‐1 cells treated with 50 mg/L IS for 72 h, where actin filaments changed their organization compared with control cells, resulting in prominent parallel‐oriented stress fibres. Dissolution of the dense peripheral band and appearance of stress fibres can arise from exposure to stresses, such as shear stress or oxidative stress. These events are in turn associated with an increase in endothelium permeability (Ogunrinade et al., 2002). For this reason, because junctional proteins contribute to regulate permeability between endothelial cells, we evaluated VE‐cadherin and beta‐catenin expression by Western blot, but we found no significant differences between control and IS‐treated cells (data not shown). It might be interesting to assess the intracellular localisation of junctional proteins rather than their total amount. For instance, some authors showed by immunofluorescence continuous linear staining for p120‐catenin, VE‐cadherin and beta‐catenin at cell–cell contacts in controls and intercellular gap and discontinuous staining in BPAECs treated with IS (Peng et al., 2012). Unfortunately, our in vitro model did not allow us to replicate such an experiment, because HMEC‐1 cells never reach confluency and, when they are too numerous, they arrange themselves in more than one layer rather than forming a well‐organized monolayer.

A previous study compared protein expression in HUVECs treated with uremic serum, that is, serum from HD patients, or with normal serum from healthy subjects. Mainly, the authors found differential expression of proteins linked to inflammation, oxidative stress and cytoskeleton (Carbó et al., 2008). Clearly, the uremic serum contains all the uremic toxins. Our proteomic analysis is the first to assess differential protein expression induced by a single uremic toxin, IS, with the aim of better elucidating the effects due specifically to this uremic toxin. The volcano plot (Figure 7) show that only a few proteins were overexpressed or underexpressed after 72 h of treatment with the highest IS concentration (50 mg/L) compared with the lowest concentration (0.5 mg/L). We did not compare the cells treated with the highest concentration of IS (50 mg/L) and the control cells because endothelial cells are physiologically exposed to IS concentrations higher than 0.5 mg/L. We analysed the proteomic data with the software String, gathering proteins according to their functions, with the aim of understanding whether exposure to IS specifically altered proteins expression of specific metabolic pathways. The networks resulting from the analysis of intracellular proteins did not show specific intracellular metabolic pathways markedly altered by HMEC‐1 cell exposure to 50 mg/L IS (Figure 8). Differently, the networks resulting from the analysis of proteins released into the medium by HMEC‐1 cells exposed to the highest pathological IS concentration (50 mg/L) revealed dysregulation of pathways linked to actin filament organization (Figure 9).

Among the intracellular proteins that have been found to be most up‐regulated or down‐regulated following treatment with pathological IS concentrations, elongation factor 1‐alpha 2, ephrin type‐B receptor 6, isoform 2 of low molecular weight phosphotyrosin protein phosphatase and thrombomodulin have been linked to actin filament organization in cell models other than HMEC‐1 (Chiarugi, 2001; Hsu et al., 2012; Kurasawa et al., 1996; Murray et al., 1996; Shimizu et al., 2005; Truitt & Freywald, 2011). Therefore, these proteins significantly up‐regulated or down‐regulated could explain, at least in part, the abnormal organization of actin filaments in cells treated with IS, which are prone to form stress fibres rather than randomized arrays. Armadillo repeat‐containing protein 8 and ephrin type‐B receptor 6 are reported to lower alpha‐catenin, beta‐catenin and cadherin 17 expression, when they are, respectively, up‐regulated or down‐regulated (Gul et al., 2019; Liang et al., 2019; Suzuki et al., 2008; Tewari et al., 2010), supporting the hypothesis mentioned before that IS could modify junctional proteins expression. Moreover, these two proteins are involved in the epithelial–mesenchymal transition (EMT): specifically, armadillo repeat‐containing protein 8 acts on the TGF‐beta pathway (Liang et al., 2017), while ephrin type‐B receptor 6 modulates metalloprotease expression (Truitt & Freywald, 2011). Therefore, it would be interesting to assess whether cells exposed to pathological IS concentrations show markers of EMT.

The two most interesting proteins found to be strongly down‐regulated by HMEC‐1 cell exposure to 50 mg/L IS compared with 0.5 mg/L IS are COP9 signalosome complex subunit 9 (CSN) and thrombomodulin (TM). CSN, in addition to its main function of controlling protein degradation via the ubiquitin–proteasome system, also appears to have powerful protective activities in CVD, including cardiovascular ischaemia (Milic et al., 2019). CSN blocks inflammatory signalling in myeloid cells, regulates the cholesterol efflux pathway in foam cells, helps control the proliferation of vascular smooth muscle (VSM) cells and T cells, plays a role in adipocyte differentiation and inhibits atherogenic signalling pathways in endothelial cells (Milic et al., 2019). Because these inflammatory and signalling pathways are implicated in atherosclerotic pathogenesis, CSN dysregulation correlates with atherosclerosis. A previous study on HMEC‐1 cells showed that overexpression of CSN down‐regulated TNF‐alpha/LPS‐induced proinflammatory cytokine levels and avoided the increased endothelial permeability induced by LPS stimulation (Colgan & Taylor, 2010). In addition, some experimental evidence suggests that CSN may regulate vascular tone at several levels, involving VSM cells and endothelial cells (Martin & Wang, 2015). Overall, CSN is emerging as a potential target for several CVD, such as atherosclerosis and ischaemia, hypertension, Raynaud's disease and coronary artery spasm.

TM is known to participate in the regulation of coagulation, innate immunity, inflammation and cell trafficking. Given its multiple functions, TM plays protective or exacerbating effects in the pathogenesis and/or progression of many diseases such as cancer, diabetic nephropathy, ischaemia–reperfusion injury, idiopathic pulmonary fibrosis, asthma, preeclampsia and atherosclerosis (Loghmani & Conway, 2018). For example, TM is down‐regulated on endothelial cells that overly atherosclerotic lesions (Laszik et al., 2001) and might inhibit endothelial–mesenchymal transition, which is critical in the CVD progression, which in turn contributes to vascular calcification, hypertension, system sclerosis and organ fibrosis (Sánchez‐Duffhues et al., 2018). Reduced TM function causes thrombosis, as observed in several animal studies (Weiler & Isermann, 2003). Plasma TM level has an inverse relationship with haemorrhagic stroke (Johansson et al., 2002). TM seems to exert its anti‐inflammatory activities attenuating NF‐κB/NLRP3 pathway, reducing IL‐1β and HMGB1 release and enhancing Nrf‐2 antioxidant activity (Yang et al., 2014). TM anti‐inflammatory activities could explain, at least in part, some results obtained in preclinical studies, in which administration of soluble TM showed to have benefits in transplantation‐associated vasculopathies, to protect heart, lung and kidney from ischaemia–reperfusion injury (Loghmani & Conway, 2018) and to improve outcome in spinal injury (Taoka et al., 2000). Taken together, this evidence makes TM another potential target for therapies for CVD. Therefore, both CSN and TM could represent a link between IS and CVD development.

We can conclude that, in the HMEC‐1 cell line, IS affected cell proliferation and actin filament organization, as well as induced protein thiol oxidation in the first 24 h of treatment, subsequently restored by the activation of antioxidant pathways, such as Nrf‐2. Moreover, CSN and TM, which are strongly correlated with CVD and inflammation, were significantly down‐regulated following cell exposure to pathological IS concentration, thus suggesting their potential role in linking IS and CVD. A more in‐depth analysis could shed light at molecular level on a possible direct effect of IS on both proteins. The modest effects of IS we found in our study, often in contrast to those observed in other studies, may be due to the choice of using pathophysiological IS concentrations measured in humans. Furthermore, we added the toxin to the culture medium on the first day of the experiment and evaluated the effects after 24, 48 or 72 h, without repeating the treatment. Thus, it is possible that, over time, the cells can metabolise IS, showing limited effects at long treatment times. Further studies will be needed to better understand the toxic effects of IS. The proteomics results of our study could be a starting point from which to select up‐regulated or down‐regulated proteins with a proven link to CVD and to confirm there IS‐induced alteration also by other methods, in other cell lines and/or in vivo, by analysing them in the plasma of healthy individuals and patients with CKD.

## CONFLICT OF INTEREST

There are no known conflicts of interest associated with this publication.

## AUTHOR CONTRIBUTIONS

Graziano Colombo, Emanuela Astori and Lucia Landoni conducted the formal analysis, performed the investigation and wrote the original draft of the manuscript. Alessandra Altomare and Maria Lisa Garavaglia designed the methodology. Aldo Milzani and Isabella Dalle‐Donne supervised the study and reviewed and edited the manuscript. Daniela Giustarini, Ranieri Rossi, Maria Chiara Lionetti and Nicoletta Gagliano reviewed and edited the manuscript. Graziano Colombo acquired the funding.

## Data Availability

All data generated or analysed during this study are included in this published article.
